# Validation of the Alcohol-Related Sexual Consequences Scale in Swedish University Students

**DOI:** 10.3390/ijerph20021035

**Published:** 2023-01-06

**Authors:** Claes Andersson

**Affiliations:** Department of Criminology, Malmö University, SE-205 06 Malmoe, Sweden; claes.andersson@mau.se

**Keywords:** alcohol-related sexual consequences, college/university students, convergent/divergent validity, gender, relationship status, gender identity/sexual orientation, age

## Abstract

Background: Alcohol-related sexual consequences are common in college students. A newly developed 41-item Alcohol-Related Sexual Consequences Scale has recently been evaluated in at-risk young adults in the U.S. The current study aims to validate the Scale in Swedish college students. Methods: The occurrence of alcohol-related sexual consequences was assessed by birth gender, relationship status, gender identity/sexual orientation, and age. Negative binomial regression was used to assess convergent and divergent validity. Results: On average, 5.4 (*SD* 5.1) alcohol-related sexual consequences were experienced past three months. Greater scores were reported in singles, LGBTQ (Lesbian, Gay, Bisexual, Transgender, Queer/Questioning), and younger students. All sex-related covariates showed robust associations with alcohol-related sexual consequences while most alcohol-related covariates were not associated (e.g., convergent validity). All alcohol-related covariates showed robust associations with alcohol consequences while most sex-related covariates were not associated (e.g., divergent validity). In the full model predicting alcohol-related sexual consequences, being a woman, single, and younger were identified as independent predictors. Conclusions: This newly developed scale assessing alcohol-related sexual consequences could be used in both epidemiological studies and intervention studies targeting at-risk students.

## 1. Introduction

Alcohol use is associated with a wide range of consequences over the college years. A recent study by Glenn et al. [1] reports that U.S. college students, on average, experience 25 alcohol-related consequences each year. Among alcohol-related sexual consequences, later regretted sex was reported by 55.8%, inappropriate sexual advances towards someone else was reported by 38.5% and experiencing being pressured or forced to have sex was reported by 24.7% [1]. Alcohol-related sexual consequences are multidimensional, also including for example unprotected sex [2], sexually transmitted infections [3], unintentional pregnancy [4], and sexual dysfunction [5]. Studies suggest that alcohol-related sexual consequences may differ by gender [6], age, and relationship status, and where being older and in a committed relationship generally serves a protective factor [7,8], sexual orientation and gender identity [9], and cultural setting [10].

Frequently used measures of alcohol consequences do not capture the full range of alcohol-related sexual consequences. The Rutgers Alcohol Problem Index (RAPI) [11] does not include any items on alcohol-related sexual consequences, the Young Adult Alcohol Problems Screening Test (YAAPST) [12] includes one item, and the Young Adult Alcohol Consequences Questionnaire (YAACQ) [13] includes two items. Limited items on alcohol-related sexual consequences are also included in other measures, such as the WHO (World Health Organization) Disability Assessment Schedule (WHODAS 2.0) [14], the WHO Alcohol, Smoking and Substance Involvement Screening Test (ASSIST) [15], and the CORE Alcohol and Drug Survey [16].

To address this limitation, Fairlie et al. [17] developed and evaluated the Alcohol-Related Sexual Consequences Scale including 41 items, showing both convergent and divergent validity. A limitation of the evaluation is that it was conducted with a sample of at-risk young adults aged 18–25 years (*n* = 318), which may have biased results. Since the scale has only been evaluated in the U.S., it is unknown whether the scale is valid in other cultural settings. 

The current study validates the Alcohol-Related Sexual Consequences Scale using the same principal methodology to assess convergent and divergent validity as previously used by Fairlie et al. [17]. The objectives were to study whether the measure is valid in a Swedish sample of university students and, in relation to previous research on the relationship between alcohol-related sexual consequences and demographic variables [6,7,8,9,10], to expand the analysis by studying whether alcohol-related sexual consequences are predicted by birth gender, relationship status, gender identity, and sexual orientation, and age.

## 2. Materials and Methods

### 2.1. Participants and Procedures

The sample used for the present study includes 2147 Swedish university students assessed for eligibility in a four-armed randomized intervention study initiated in 2007 [18]. Due to eligibility criteria and the large sample size needed for the intervention study, the original invitation to participate was sent to 28,617 students, of which 3046 (10.6%) responded to an online survey including 229 questions before the intervention. 

Students were selected from university records from six universities across Sweden, including students aged 30 years or younger and studying at least half-time. Selected students were only sent one invitation by email, and to increase response rates, respondents were included in a lottery arranged by the no-profit charitable fund Save the Children. Prizes in the lottery included 33 gift vouchers valued between SEK 500–10,000 (approximately USD 50-1000). The emails were an integrated feature of an assessment and intervention platform specifically developed for the present study.

For the present analysis, among respondents assessed for eligibility in the intervention study, 899 (29.5%) were excluded for the following reasons: not drinking alcohol over the past three months (*n* = 245); no sexual experience over the past three months (*n* = 456); not drinking alcohol or having a sexual experience over the past three months (*n* = 198).

In the present sample, utilizing the same criteria as in the study conducted by Fairlie et al. [13] would have resulted in 214 (7%) eligible students, e.g., 2832 (93%) would have been excluded from the current study. 

To reduce the response burden for participants, only a limited number of demographic variables were included in the current survey (see Table 1). Compared to official records on Swedish university students [19], the present sample comprises a somewhat younger population and a greater proportion of women.

### 2.2. Measures

Alcohol-related sexual consequences were assessed by using the Alcohol-Related Sexual Consequences Scale [17]. Participants indicated whether they had experienced each of the 41 negative consequences because of drinking in the past three months (0 = no, 1 = yes). The questions included in the form appear in Table A1. The remaining measures used in the present study are partly different from the study conducted by Fairlie et al. [17]. 

Demographic information in the present study included birth gender (0 = male, 1 = female), relationship status (0 = single, 1 = attached in a committed relationship), gender identity and sexual orientation (0 = straight, 1 = LGBTQ, Lesbian, Gay, Bisexual, Transgender, Queer/Questioning), and age which is handled both as a continuous variable and as a dichotomized variable (0 = 18–24 years, 1 = 25–30 years). In the U.S. study [17], only age and gender are used for the primary analysis. 

Four alcohol-related variables were used in the analysis: Participants were asked to estimate the number of standard drinks they usually have had on a drinking occasion over the past three months. Alcohol expectancies were assessed using the Brief Comprehensive Effects of Alcohol Scale [20]. The original scale includes 15 items and responses on each item ranges from 0 (disagree) to 3 (agree). For the present study were two items assessing sexual expectancies eliminated, resulting in a 13-items scale. Mean scores were computed for a 6-item negative expectancy subscale (alpha = 0.662) and a 7-item positive expectancy subscale (alpha =0.792). The Brief Young Adult Alcohol Consequences Questionnaire [21] was used as an alternative consequence variable. The measure includes 24 alcohol-related consequences and where it is indicated whether each consequence occurred over the past three months (0 = no, 1 = yes). For the present study, one item assessing sex-related consequences was excluded, resulting in a 23-items summa score. Instead of drinks per occasion, the U.S. study [17] used total drinks per week, while the remaining instruments are the same.

Four sex-related variables were included in the analysis: Participants reported the number of standard drinks consumed, on average, before or during any sex over the past three months. Sex-related alcohol expectancies were assessed using a scale developed by Dermen and Cooper [22]. The scale includes 13 items and responses on each item ranges from 0 (strongly disagree) to 5 (strongly agree). Mean scores were computed for a 5-item sexual enhancement expectancies subscale (alpha = 0.853), a 4-item sexual risk-taking expectancies subscale (alpha = 0.857), and a 4-item sexual disinhibition expectancies subscale (alpha 0.840). The U.S. study [17] also included the number of casual partners, and the number of times drinking alcohol before/during sex with a casual partner.

### 2.3. Analysis

The principal analytic strategy is based on Fairlie et al. [17], though it should be noted that the U.S. study analyzed data in three steps while the present study utilizes a four-step procedure. As in the evaluation conducted by Fairlie et al. [17], models were designed to demonstrate convergent validity (i.e., robust associations between alcohol-related consequences and sex-related covariates) and divergent validity (i.e., robust associations between alcohol consequences and alcohol-related covariates) [23].

Mean scores and standard deviations (*SD*) on the alcohol-related sexual consequences scale were calculated for the total sample and by demographic variables, whereafter pairwise comparisons were made using the Mann–Whitney U-test. Chi-square tests were conducted for each of the 41 items to examine differences by demographic variables as a supplementary analysis. A Bonferroni correction was used with an α of *p* < 0.0003 for chi-square tests. Raw values are reported for all variables in the regression models, and as supplementary analysis, Spearman’s Rho correlations were calculated. Since the primary study outcomes were over-dispersed count variables, negative binomial regression was used. Four-step hierarchical models were conducted separately for two outcomes: the number of alcohol-related sexual consequences and the number of alcohol consequences. In Step 1, which is not included in the study by Fairlie et al. [17], four demographic predictors were included in the analysis: Birth gender; Relationship status; Gender identity/sexual orientation; age. The rationale for adding this step is related to the specific objectives of the present study, e.g., to investigate whether alcohol-related sexual consequences are predicted by these demographic variables. In Step 2, three alcohol-related predictors were included in the analysis: Drinks per occasion; Negative alcohol expectancies; Positive alcohol expectancies. In Step 3, four sex-related predictors were included in the analysis: Drinks before or during sex; Sexual enhancement expectancies; Sexual risk-taking expectancies; Sexual disinhibition expectancies. In Step 4, the alternative consequences measure was added. SPSS version 28.0.1.1(15) was used in all statistical analyses (IBM Corp., Armonk, NY, USA).

## 3. Results

### 3.1. Descriptive Statistics

Shown in Table 1 are group comparisons of alcohol-related sexual consequences over the past three months assessed on the newly established 41-item measure. Mean scores were greater in students being single compared to students attached in a committed relationship, in LGBTQ students compared to straight students, and in younger students compared to older students. As reported in the study by Fairlie et al. [13], there was no significant difference related to birth gender. The U.S. study only included students aged 18–25 and no comparisons were made by age, relationship status, and sexual identity/sexual orientation. 

Table A1 presents responses on individual items. In all groups, the most frequently reported consequences referred to protection (oral or vaginal sex without condom/dental dam). Being unable to climax was among the five most prevalent consequences in all groups. Men reported neglect to use birth control other than a condom, and oral and anal sex with someone I had just met to a greater extent than women, and women reported being unable to climax and worry about pregnancy to a greater extent than men. In the U.S. study by Fairlie et al. [17], men were more likely than women to report oral sex with someone they just met. In the current study, students being single reported a significantly greater proportion than attached students on the following twenty-one items: Digital/Oral/Vaginal sex later regretted; Relationship issues with a romantic partner; Got a bad reputation; Neglected use of birth control other than a condom; Had sex with my partner without talking about using birth control other than a condom; Unable to climax; Digital/Oral/Vaginal/Anal sex with someone I wouldn’t have when sober; Digital/Oral/Vaginal/Anal sex with someone I had just met; In a sexual situation that I wouldn’t have been in if I was sober; In a sexual situation in which I felt unsafe; Had sex and acquired a sexually transmitted infection (STI); Had sex and was scared that I acquired an STI; Someone fondled, etc. or removed clothes without my consent. LGBTQ students reported anal sex with someone I had just met to a greater extent than straight students. Younger students (18–24 years) reported a significantly greater proportion than older students (25–30 years) in the following six items: Vaginal sex later regretted; Relationship issues with a romantic partner; Unable to climax; Vaginal sex with someone I had just met; In a sexual situation that I wouldn’t have been in if I was sober; Had sex and was worried about pregnancy. 

Table A2 presents a comparison of alcohol-related sexual consequences reported during the past three months in Sweden and alcohol-related sexual consequences reported during the past month in the U.S. study that was conducted by Fairlie et al. [13]. U.S. participants report greater frequencies than the Swedish participants on thirty out of the forty-one variables.

Descriptive statistics for the variables included in the regression analysis are shown in Table 2, and correlations are shown in Table A3. The number of alcohol-related sexual consequences and the number of alcohol-related consequences were correlated (*r* = 0.531). By computing the *R*^2^, only 28.2% of the variance was shared between measures. Participants reported consuming 4.02 (*SD* 3.42) standard drinks on a regular occasion and 2.63 (*SD* 2.85) standard drinks when drinking before or during sex. In comparison, Fairlie et al. [17] did not report the number of standard drinks on a regular occasion, but it was reported that students consume on average 4.98 (2.8) drinks when drinking before or during sex.

### 3.2. Prediction of Alcohol-Relates Sexual Consequences

The demographic variables were entered in Step 1 of the negative binomial regression model prediction of alcohol-related sexual consequences (Table 3). Only being single and being a younger student was associated with the outcome. Alcohol-related variables were entered in Step 2. Drinks per occasion and positive alcohol expectancies were positively associated with the outcome. Among the demographic variables, being single and being a younger student remained associated. Sex-related variables were added to the model in Step 3. Drinks before or during sex, sexual enhancement expectancies, sexual risk-taking expectancies, and sexual disinhibition expectancies were all positively associated with alcohol-related consequences. The alcohol-related variables entered at the previous step were no longer significant. Among the demographic predictors, being single and being a younger student remained associated. Additionally, being a woman was now positively associated with the outcome. In Step 4, alcohol consequences were entered and found positively associated with alcohol-related sexual consequences. All sex-related variables remained significant predictors. Among the alcohol-related variables, only negative alcohol expectancies appeared to be negatively associated with the outcome. Being a woman, single, and a younger student remained significant predictors of alcohol-related sexual consequences. Though somewhat different variables and analytic procedures were used in the preceding study by Fairlie et al. [17], a similar pattern was identified. However, it should be noted that the number of drinks before/during sex and sexual enhancement expectancies were not significant in the final step of the U.S. study.

### 3.3. Prediction of Alcohol Consequences

The same procedure was utilized to predict the alternative consequence variable, e.g., alcohol consequences (Table 4). In Step 1, the outcome was associated with male birth sex, being single, and being a younger student. In Step 2, drinks per occasion, negative alcohol expectancies, and positive alcohol expectancies were all positively associated with the outcome. Among the demographic predictors, being male, single and a younger student remained associated. In Step 3, sex-related predictors were entered, and of which only drinks before or during sex, and sexual disinhibition expectancies were associated with the outcome. All alcohol-related variables remained significant, and so did being single and being a younger student but being of male birth gender was no longer significant. In Step 4, alcohol-related sexual consequences exhibited a positive association with alcohol consequences. All alcohol-related variables remained significant predictors. Drinks before or during sex and sexual disinhibition expectancies, as well as being single, remained positively associated with alcohol consequences. Once again, the overall pattern was similar in the study by Fairlie et al. [17] where most alcohol-related variables and sexual disinhibition expectancies were significant in the final step, but where drinks before/during sex were not significant in the final step.

## 4. Discussion

The present study presents a validation of a new measure of alcohol-related sexual consequences in a Swedish setting, using the same principal methodology to assess convergent and divergent validity as previously used by Fairlie et al. [17] in U.S. students. In a diverse sample of Swedish university students, all sex-related covariates showed robust associations with alcohol-related sexual consequences while most alcohol-related covariates were not associated (e.g., convergent validity). All alcohol-related covariates showed robust associations with alcohol consequences while most sex-related covariates were not associated (e.g., divergent validity). The overall findings on validity correspond to what previously has been reported in an at-risk sample of U.S. students [17].

In the full models in the current study, three non-demographic predictors were associated with both alcohol-related sexual consequences and alcohol consequences. First, negative alcohol expectancies had a negative association with alcohol-related sexual consequences but a positive association with alcohol consequences. Since negative alcohol expectancies are known to have a positive association with greater negative alcohol consequences [24], the negative association identified in the current study underlines that alcohol consequences and alcohol-related sexual consequences are different constructs. Second, the quantity of alcohol consumed before or during sex was positively associated with both outcomes, while overall quantity per occasion only had a positive association with alcohol consequences. This is because alcohol consumption increases the risk of both alcohol-related sexual consequences and alcohol consequences [12]. Third, as in the study by Fairlie et al. [17], sexual disinhibition expectancies were associated with both alcohol-related sexual consequences and alcohol consequences. The reason for this may be a general association between disinhibition expectancies and problem drinking [25]. The current study confirms findings from the U.S. study conducted by Fairlie et al. [17], which uses similar procedures as in the current study but in a highly selected sample of at-risk young adults, e.g., the eligibility criteria in the U.S. study selected at-risk students, while the present study includes all sexually active and alcohol consuming students.

The binomial regression models showed that being a woman was an independent predictor of alcohol-related sexual consequences after controlling for actual alcohol use and expectancies. There were no differences on the total scale, and the adjusted Chi-square tests only found that women reported being unable to climax and worry about pregnancy to a greater extent than men. A similar finding has not been reported in previous studies using the same measure in at-risk populations [17,18,26,27], nor in reports on gender differences using measures including items on alcohol-related sexual consequences [28]. However, it has been reported that the consequences of heavy alcohol use appear to be more negative for women than men in relation to cognitive and motor impairment, physical harm, sexual assault, and reproductive problems [6] and that both biological factors and psycho-socio-cultural factors explain gender differences [29]. Gender differences in alcohol-related sexual consequences remain to be further investigated. 

The regression models also showed that being single and being a younger student predicted alcohol-related sexual consequences. Being single was also an independent predictor of alcohol consequences. These two groups have been identified as risk groups for heavy drinking [30] and with high rates of alcohol-related sexual consequences [13]. Previous research has identified sexual minorities as a risk group for heavy drinking [31] and sexual assault [8]. In the current study, LGBTQ students reported greater scores on the alcohol-related sexual consequences scale, but the sexual minority group was not identified as a significant predictor in any of the regression models. 

When comparing the current frequencies of alcohol-related sexual consequences with the preceding U.S. study [17], it should be noted that U.S. participants were asked to report consequences over one month and Swedish participants were asked to report them over three months. Despite this, the U.S. participants report greater frequencies than the Swedish participants on thirty out of the forty-one variables (see Table A2). A likely reason for this is that the U.S. sample included at-risk young adults, whereas the Swedish sample includes a less selected sample. Two of the items where the Swedish sample reported greater frequencies are “Had sex and become (or got my partner) unintentionally pregnant” and “Had sex and acquired a sexually transmitted infection (STI)”, which are consequences likely to be more apparent to the respondent after three months than after one month. Likely, such consequences are better captured by utilizing a three-month timeframe. By repeating the U.S. study in a Swedish sample, and by including a more diverse sample of university students, the present study shows the validity of the scale in a different cultural setting and in a sample that, relative to the study conducted by Fairlie et al. [17], is likely to be more representative of students since only abstainers were excluded in the current study. If the same at-risk eligibility criteria would have been applied in the Swedish sample, 93% of the sample would have been excluded.

Even if the scale offers an extension in relation to other available measures of alcohol-related sexual consequences, there are some limitations. Fairlie et al. [17] discuss that not all aspects of alcohol-related sexual behaviors are included, that the measure does not assess experiences happening while drinking, and that the measure is only relevant to individuals who both drink alcohol and have sex. It should also be noted that the scale is extensive and that several of the individual items are lengthy, leading to a considerable burden for respondents. Among the limitations of the current study, it should be noted that only 10.6% of the students invited responded, which may have biased results. One limitation concerns that the use of somewhat different measures and the use of somewhat different analytic procedures limit direct comparisons between the current study and the preceding U.S. study [17]. A direct comparison was not possible, and the current objective was to re-evaluate the measure in a more diverse sample, and by using available measures. To simplify comparisons between the two studies, comments on differences and similarities have been made where possible throughout the text. Moreover, in the present study, only a few demographic variables were collected to reduce the response burden for participating students, which limits the possibility to analyze representativity in relation to publicly available statistics. Based on available data, female students and younger students are somewhat overrepresented compared to official university records. It should be noted that women generally are more likely to respond to surveys [32] and that students older than 30 years and not studying full-time were excluded from the sample selected from university records. 

The current study indicates that the scale may also be useful in epidemiological studies on alcohol-related sexual consequences. If necessary to reduce the response burden for participants in such studies, the four items assessing digital, oral, vaginal, and anal sex separately may be collapsed, decreasing the scale to 29 items. The evaluated measure has previously been shown to be useful in personalized feedback intervention targeting alcohol-related sexual behaviors in at-risk students [19,26,27]. 

## 5. Conclusions

The newly developed Alcohol-Related Sexual Consequences Scale shows convergent and divergent validity in Swedish university students. In a Swedish setting, the scale could be used to identify alcohol-related sexual consequences in both epidemiological studies and intervention studies targeting at-risk students.

Alcohol-related sexual consequences were found to be associated with being a woman, single, and younger student. The overall frequencies indicate that alcohol-related sexual consequences are a common problem among Swedish university students, emphasizing the need for the development and implementation of effective interventions.

## Figures and Tables

**Table 1:** By demographic subgroup, comparison of total scores on the Alcohol-Related Sexual Consequences Scale.

		Alcohol-Related Sexual Consequences		
		*n*	Mean (*SD*)	*p*
Birth gender	Men	577	5.53 (4.99)	0.767
	Women	1570	5.49 (5.31)	
Relationship status	Single	538	7.71 (6.02)	0.000
	Attached	1609	4.76 (4.49)	
Gender identity/sexual orientation	Straight	1804	5.40 (5.07)	0.021
	LGBTQ	343	5.98 (5.10)	
Age	18–24	1256	5.99 (5.29)	<0.001
	25–30	891	4.80 (4.67)	

**Table 2:** Descriptive statistics for variables in the binomial regression models.

	*n*	Range	M (*SD*) or %
Dependent Variable/Alternative Consequence Variable			
Alcohol-related sexual consequences	2147	0–37	5.40 (5.08)
Alcohol Consequences	2147	0–24	5.85 (4.45)
Demographic variables			
Female sex	2147	0–1	73.1%
Single	2147	0–1	25.1%
LGBTQ	2147	0–1	16.0%
Age	2147	18–30	24.02 (2.85)
Alcohol-related variables			
Drinks per occasion	2147	0–25	4.02 (3.42)
Negative alcohol expectancies	2147	0–18	8.21 (2.55)
Positive alcohol expectancies	2147	0–21	12.43 (3.01)
Sex-related variables			
Drinks before/during sex	2147	0–25	2.63 (2.85)
Sexual enhancement expectancies	2147	0–25	7.14 (5.42)
Sexual risk-taking expectancies	2147	0–20	7.58 (5.56)
Sexual disinhibition expectancies	2147	0–20	7.84 (5.33)

**Table 3:** Results of negative binomial regression models predicting alcohol-related sexual consequences.

	Step 1			Step 2			Step 3			Step 4		
Predictor	*B*	*SE*	*p*	*B*	*SE*	*p*	*B*	*SE*	*p*	*B*	*SE*	*p*
Demographic variables												
Female sex	0.014	0.0487	0.778	0.085	0.0482	0.079	0.170	0.0459	<0.001	0.198	0.0426	<0.001
Single	0.463	0.0492	0.000	0.387	0.0484	<.001	0.215	0.0460	<0.001	0.148	0.0425	<0.001
LGBTQ	0.046	0.0586	0.438	0.057	0.0571	0.321	0.067	0.0533	0.207	0.061	0.0491	0.212
Age	−0.031	0.0077	<.001	−0.019	0.0075	0.012	−0.020	0.0070	0.004	−0.016	0.0065	0.013
Alcohol-related variables												
Drinks per occasion				0.047	0.0069	<0.001	0.009	0.0064	0.151	−0.008	0.0059	0.156
Negative alcohol expectancies				0.014	0.0085	0.105	0.001	0.0076	0.771	−0.030	0.0079	<0.001
Positive alcohol expectancies				0.044	0.0072	<0.001	0.001	0.0076	0.877	−0.006	0.0071	0.401
Sex-related variables												
Drinks before/during sex							0.084	0.0084	0.000	0.055	0.0078	<0.001
Sexual enhancement expectancies							0.015	0.0042	<0.001	0.013	0.0039	<0.001
Sexual risk-taking expectancies							0.013	0.0042	0.001	0.011	0.0039	0.003
Sexual disinhibition expectancies							0.030	0.0050	<0.001	0.016	0.0047	<0.001
Alternative consequence variable												
Alcohol Consequences										0.082	0.0049	0.000
Other parameters												
Intercept	2.294	0.1940	0.000	1.091	0.2266	<0.001	1.207	0.2119	<0.001	1.180	0.1948	<0.001
Dispersion	0.794	0.034		0.730	0.0324		0.594	0.0286		0.467	0.0247	

**Table 4:** Results of negative binomial regression models predicting alcohol consequences.

	Step 1			Step 2			Step 3			Step 4		
Predictor	*B*	*SE*	*p*	*B*	*SE*	*p*	*B*	*SE*	*p*	*B*	*SE*	*p*
Demographic variables												
Female sex	−0.155	0.0383	<0.001	−0.084	0.0351	0.016	−0.025	0.0338	0.465	−0.059	0.0318	0.061
Single	0.355	0.0389	0.000	0.259	0.0354	<0.001	0.148	0.0342	<0.001	0.082	0.0322	0.011
LGBTQ	0.066	0.0467	0.155	0.063	0.0422	0.137	0.040	0.0399	0.316	0.038	0.0374	0.307
Age	−0.028	0.0062	<0.001	−0.012	0.0056	0.039	−0.012	0.0053	0.022	−0.006	0.0050	0.212
Alcohol-related variables												
Drinks per occasion				0.063	0.0051	0.000	0.038	0.0049	<0.001	0.035	0.0046	<0.001
Negative alcohol expectancies				0.056	0.0064	0.000	0.046	0.0063	<0.001	0.043	0.0059	<0.001
Positive alcohol expectancies				0.057	0.0055	0.000	0.029	0.0058	<0.001	0.032	0.0055	<0.001
Sex-related variables												
Drinks before/during sex							0.054	0.0059	0.000	0.032	0.0056	<0.001
Sexual enhancement expectancies							0.006	0.0032	0.062	0.002	0.0030	0.548
Sexual risk-taking expectancies							0.003	0.0032	0.398	−0.001	0.0030	0.640
Sexual disinhibition expectancies							0.027	0.0037	<0.001	0.019	0.0035	<0.001
Alternative consequence variable												
Alcohol-related sexual consequences										0.047	0.0030	0.000
Other parameters												
Intercept	2.444	0.1551	0.000	0.536	0.1684	0.001	0.631	0.1598	<0.001	0.434	0.1506	0.004
Dispersion	0.449	0.0208		0.321	0.0170		0.263	0.0152		0.207	0.0133	

## Data Availability

The data that support the findings of this study are available from the corresponding author, upon reasonable request.

## Appendix A

**Table A1:** In total and by demographic subgroup, responses, and comparisons on individual items on the Alcohol-Related Sexual Consequences Scale.

		Total (%)	Birth Gender				Relationship Status				Gender Identity/Sexual Orientation				Age			
			Men (%)	Women (%)			Single (%)	Attached (%)			Straight (%)	LGBTQ (%)			18–24 yrs (%)	25–30 yrs (%)		
		*n* = 2147	*n* = 577	*n* = 1570	*X* ^2^	*p*	*n* = 538	*n* = 1609	*X* ^2^	*p*	*n* = 1804	*n* = 343	*X* ^2^	*p*	*n* = 1256	*n* = 891	*X* ^2^	*p*
1	Digital sex later regretted	4.8	5.9	4.5	1.882	0.170	11.2	2.7	61.982	<0.001 *	4.6	6.1	1.448	0.229	6.1	3.1	9.566	0.002
2	Oral sex later regretted	4.7	4.5	4.8	0.069	0.793	10.0	2.9	45.544	<0.001 *	4.5	5.5	0.635	0.426	5.7	3.3	7.138	0.008
3	Vaginal sex later regretted	8.2	7.6	8.3	0.291	0.590	15.4	5.7	50.773	<0.001 *	8.4	6.7	1.139	0.286	10.0	5.6	13.117	<0.001 *
4	Anal sex later regretted	2.0	1.6	2.2	0.942	0.332	3.3	1.6	6.010	0.014	1.9	2.6	0.671	0.413	1.9	2.2	0.289	0.591
5	Cheated on a romantic partner	3.6	3.5	3.7	0.063	0.802	5.4	3.0	6.333	0.012	3.0	6.7	11.008	<0.001	4.1	3.0	1.580	0.209
6	Relationship issues with a romantic partner	21.8	19.1	22.8	3.459	0.063	14.7	24.2	21.312	<0.001 *	22.0	21.0	0.156	0.698	24.6	17.8	13.960	<0.001 *
7	Got a bad reputation	2.7	4.2	2.1	6.912	0.009	5.9	1.6	30.124	<0.001 *	2.4	3.8	2.035	0.154	3.1	2.0	2.374	0.123
8	Neglected to use birth control other than a condom	11.7	17.0	9.7	21.418	<0.001 *	17.8	9.6	26.327	<0.001 *	10.8	16.3	8.497	0.004	13.1	9.8	5.475	0.019
9	Had sex with my partner without talking about using birth control other than a condom	21.4	23.4	20.6	1.912	0.167	31.4	18.0	43.001	<0.001 *	21.1	22.7	0.450	0.502	22.5	19.9	2.075	0.150
10	Had sex with my partner without talking about condom use	43.4	39.3	44.9	5.315	0.021	45.7	42.6	1.567	0.211	43.5	43.1	0.011	0.915	44.9	41.3	2.754	0.097
11	Oral sex without a condom	64.7	66.6	64.1	1.132	0.287	68.6	63.5	4.652	0.031	64.7	65.0	0.013	0.908	66.8	61.8	5.615	0.018
12	Oral sex without a dental dam	58.9	62.4	57.6	4.035	0.045	61.9	57.9	2.710	0.100	58.7	59.8	0.135	0.714	61.8	54.8	10.589	0.001
13	Vaginal sex without a condom	63.4	61.0	64.3	2.012	0.156	61.5	64.1	1.133	0.287	64.7	56.6	8.325	0.004	66.2	59.5	10.264	0.001
14	Anal sex without a condom	18.2	18.9	18.0	0.244	0.621	19.7	17.7	1.072	0.301	17.8	20.1	0.995	0.319	18.0	18.5	0.096	0.756
15	Sex without a condom even though the partner wanted to use one	1.4	2.6	0.9	9.237	0.002	1.5	1.3	0.100	0.752	1.4	1.2	0.104	0.747	1.6	1.0	1.326	0.249
16	Unable to lubricate (or attain an erection)	21.1	19.6	21.7	1.154	0.283	24.3	20.1	4.419	0.036	20.3	25.7	4.980	0.026	22.5	19.3	3.098	0.078
17	Unable to climax	37.0	29.3	39.8	20.035	<0.001 *	49.3	32.9	46.411	<0.001 *	36.4	39.9	1.535	0.215	40.8	31.6	18.580	<0.001 *
18	Digital sex with someone I wouldn’t have when sober	6.3	7.3	6.0	1.187	0.276	14.5	3.6	80.639	<0.001 *	5.9	8.5	3.093	0.079	7.8	4.3	10.995	<0.001
19	Oral sex with someone I wouldn’t have sex with when sober	5.7	6.4	5.4	0.785	0.376	13.8	3.0	87.283	<0.001 *	5.4	7.0	1.317	0.251	6.1	5.1	1.135	0.287
20	Vaginal sex with someone I wouldn’t have sex with when sober	8.2	6.6	8.7	2.582	0.108	17.3	5.1	80.025	<0.001 *	8.0	9.0	.429	0.512	9.7	5.9	9.869	0.002
21	Anal sex with someone I wouldn’t have sex with when sober	1.1	1.2	1.0	0.150	0.699	2.6	0.6	15.877	<0.001 *	0.9	1.7	1.771	0.183	0.9	1.3	1.091	0.296
22	Digital sex with someone I had just met	14.5	18.7	13.0	11.130	<0.001	34.9	7.7	240.834	<0.001 *	13.7	18.7	5.598	0.018	16.2	12.1	7.127	0.008
23	Oral sex with someone I had just met	13.4	19.1	11.3	22.111	<0.001 *	34.9	6.2	288.602	<0.001 *	12.3	19.0	10.987	<0.001	14.9	11.2	6.046	0.014
24	Vaginal sex with someone I had just met	15.2	14.7	15.4	0.152	0.696	37.4	7.8	272.313	<0.001 *	15.1	15.7	0.083	0.773	17.9	11.4	16.880	<0.001 *
25	Anal sex with someone I had just met	2.8	5.2	1.9	16.797	<0.001 *	8.4	0.9	81.981	<0.001*	2.1	6.7	22.985	<0.001*	2.8	2.8	0.001	0.979
26	In a sexual situation that I wouldn’t have been in if I was sober	17.5	20.6	16.4	5.287	0.021	37.9	10.7	206.922	<0.001 *	16.4	23.6	10.523	0.001	20.1	13.9	13.633	<0.001 *
27	In a sexual situation in which I felt unsafe	6.4	4.2	7.3	6.749	0.009	11.2	4.8	26.646	<0.001 *	6.1	8.2	2.045	0.153	8.0	4.3	11.845	<0.001
28	Went further sexually than my partner wanted to	2.2	3.6	1.7	7.115	0.008	3.2	1.9	2.805	0.094	2.0	3.5	2.979	0.084	3.0	1.1	8.638	0.003
29	Went further sexually than what I am usually comfortable with when sober	12.0	11.3	12.2	0.372	0.542	14.5	11.1	4.354	0.037	11.9	12.5	0.124	0.725	13.2	10.2	4.462	0.035
30	Had sex and became (or got my partner) unintentionally pregnant	2.0	1.7	2.1	.0292	0.589	2.0	2.0	0.006	0.936	2.1	1.7	0.134	0.715	2.1	1.8	0.333	0.564
31	Had sex and was worried about pregnancy	20.5	14.2	22.9	19.364	<0.001 *	25.1	19.0	9.117	0.003	20.5	20.7	0.006	0.936	24.6	14.8	30.591	<0.001 *
32	Had sex and acquired a sexually transmitted infection (STI)	3.2	2.6	3.4	0.957	0.328	5.9	2.3	17.254	<0.001 *	3.0	4.1	0.988	0.320	3.7	2.6	1.958	0.1562
33	Had sex and was scared that I acquired an STI	15.6	15.6	15.5	0.001	0.974	36.2	8.6	233.909	<0.001 *	15.2	17.2	0.840	0.359	17.5	12.8	8.845	0.003
34	Had sex in exchange for money or goods (i.e., drugs, rent)	0.7	1.4	0.4	5.381	0.020	1.5	0.4	6.431	0.011	0.7	0.9	0.182	0.669	0.6	0.9	0.871	0.351
35	Someone fondled etc. or removed clothes without my consent	5.1	3.5	5.7	4.248	0.039	8.4	4.0	16.100	<0.001 *	5.0	5.5	0.181	0.670	6.3	3.4	9.240	0.002
36	Someone had oral sex with me without my consent when I was too drunk	1.3	1.4	1.2	0.106	0.745	2.4	0.9	7.763	0.005	1.1	2.0	2.017	0.156	1.8	0.6	5.948	0.015
37	Forced sexual contact (with vagina/penis/anus) without my consent when I was too drunk	2.2	1.9	2.4	0.391	0.532	3.9	1.7	9.135	0.003	2.1	2.9	0.863	0.353	2.7	1.6	3.076	0.079
38	Sexual contact (with the anus) without my consent when I was too drunk	0.7	1.0	0.5	1.832	0.176	0.7	0.6	0.093	0.761	0.6	1.2	1.665	0.197	0.9	0.3	2.338	0.126
39	Some tried to have oral sex with me without my consent when I was too drunk	1.4	1.2	1.4	0.112	0.738	2.4	1.0	6.118	0.013	1.2	2.0	1.459	0.227	1.7	0.9	2.344	0.126
40	Someone tried to have sexual contact (with the vagina/penis/anus) without my consent when I was too drunk	2.2	2.1	2.3	0.088	0.767	3.7	1.7	7.212	0.007	2.2	2.6	0.281	0.596	2.8	1.5	4.203	0.040
41	A man tried to put his penis into my anus, or someone tried to stick fingers or objects into my anus without my consent when I was too drunk	0.5	0.7	0.4	0.881	0.348	0.7	0.4	1.194	0.274	0.5	0.3	0.267	0.605	0.6	0.2	1.913	0.167

* Bonferroni adjustment.

## Appendix B

**Table A2:** A comparison of alcohol-related sexual consequences reported during the past three months in Sweden and alcohol-related sexual consequences reported during the past month in the U.S. study that was conducted by Fairlie et al. [13].

		Swedish Sample	U.S. Sample
		*n* = 2147	*n* = 318
1	Digital sex later regretted	4.8	8.0
2	Oral sex later regretted	4.7	15.0
3	Vaginal sex later regretted	8.2	22.0
4	Anal sex later regretted	2.0	5.0
5	Cheated on a romantic partner	3.6	8.0
6	Relationship issues with a romantic partner	21.8	14.0
7	Got a bad reputation	2.7	12.0
8	Neglected to use birth control other than a condom	11.7	21.0
9	Had sex with my partner without talking about using birth control other than a condom	21.4	33.0
10	Had sex with my partner without talking about condom use	43.4	56.0
11	Oral sex without a condom	64.7	66.0
12	Oral sex without a dental dam	58.9	52.0
13	Vaginal sex without a condom	63.4	66.0
14	Anal sex without a condom	18.2	15.0
15	Sex without a condom even though the partner wanted to use one	1.4	9.0
16	Unable to lubricate (or attain an erection)	21.1	17.0
17	Unable to climax	37.0	30.0
18	Digital sex with someone I wouldn’t have when sober	6.3	8.0
19	Oral sex with someone I wouldn’t have sex with when sober	5.7	13.0
20	Vaginal sex with someone I wouldn’t have sex with when sober	8.2	19.0
21	Anal sex with someone I wouldn’t have sex with when sober	1.1	3.0
22	Digital sex with someone I had just met	14.5	12.0
23	Oral sex with someone I had just met	13.4	22.0
24	Vaginal sex with someone I had just met	15.2	23.0
25	Anal sex with someone I had just met	2.8	6.0
26	In a sexual situation that I wouldn’t have been in if I was sober	17.5	39.0
27	In a sexual situation in which I felt unsafe	6.4	6.0
28	Went further sexually than my partner wanted to	2.2	6.0
29	Went further sexually than what I am usually comfortable with when sober	12.0	18.0
30	Had sex and became (or got my partner) unintentionally pregnant	2.0	1.0
31	Had sex and was worried about pregnancy	20.5	20.0
32	Had sex and acquired a sexually transmitted infection (STI)	3.2	2.0
33	Had sex and was scared that I acquired an STI	15.6	14.0
34	Had sex in exchange for money or goods (i.e., drugs, rent)	0.7	3.0
35	Someone fondled etc. or removed clothes without my consent	5.1	9.0
36	Someone had oral sex with me without my consent when I was too drunk	1.3	3.0
37	Forced sexual contact (with vagina/penis/anus) without my consent when I was too drunk	2.2	3.0
38	Sexual contact (with the anus) without my consent when I was too drunk	0.7	2.0
39	Some tried to have oral sex with me without my consent when I was too drunk	1.4	4.0
40	Someone tried to have sexual contact (with the vagina/penis/anus) without my consent when I was too drunk	2.2	5.0
41	A man tried to put his penis into my anus, or someone tried to stick fingers or objects into my anus without my consent when I was too drunk	0.5	2.0

## Appendix C

**Table A3:** Bivariate correlations for the variables included in the binomial regression analysis.

		1.	2.	3.	4.	5.	6.	7.	8.	9.	10.	11.	12.
1.	Alcohol-related sexual consequences												
2.	Alcohol Consequences	0.531 **											
3.	Female sex	0.006	−0.092 **										
4.	Single	0.241 **	0.236 **	−0.066 **									
5.	LGBTQ	0.050 *	0.037	0.052 *	0.117 **								
6.	Age	0.123 **	0.123 **	0.037	0.077 **	−0.012							
7.	Drinks per occasion	0.302 **	0.473 **	−0.145 **	0.219 **	−0.027	0.149 **						
8.	Negative alcohol expectancies	0.073 **	0.225 **	0.059 **	−0.005	0.039	0.040	0.052 *					
9.	Positive alcohol expectancies	0.214 **	0.328 **	−0.075 **	0.117 **	0.015	0.101 **	0.220 **	0.165 **				
10.	Drinks before/during sex	0.468 **	0.433 **	−0.053 *	.215 **	0.017	0.082 **	0.491 **	−0.025	0.229 **			
11.	Sexual enhancement expectancies	0.283 **	0.268 **	0.003	0.166 **	0.012	0.043 *	0.167 **	0.071 **	0.436 **	0.277 **		
12.	Sexual risk-taking expectancies	0.251 **	0.267 **	−0.096 **	0.137 **	0.022	−0.001	0.157 **	0.262 **	0.252 **	0.14 5**	0.286 **	
13.	Sexual disinhibition expectancies	0.323 **	0.396 **	−0.191 **	0.207 **	0.057 **	0.014	0.237 **	0.26 8**	0.417 **	0.265 **	0.470 **	0.562 **

** Correlation is significant at the 0.01 level (2-tailed). * Correlation is significant at the 0.05 level (2-tailed).
